# Downregulation of HDGF inhibits tumorigenic phenotypes of hypopharyngeal squamous cell carcinoma by suppressing the AKT/mTOR/VEGF pathway

**DOI:** 10.3389/fonc.2025.1683145

**Published:** 2025-11-07

**Authors:** Feilong Yang, Qiang Zhang, Jiahao Shan, Xinyue Du, Yang Han, Ziyang Liu

**Affiliations:** 1 Department of Urology, General Hospital of Ningxia Medical University, Yinchuan, China; 2 School of First Clinical Medicine, Ningxia Medical University, Yinchuan, China; 3 Department of Urology, Yueyang Hospital of Integrated Traditional Chinese and Western Medicine, Shanghai University of Traditional Chinese Medicine, Shanghai, China; 4 Department of Clinical Laboratory, Inner Mongolia Xingan League People’s Hospital, Ulanhot, China

**Keywords:** HDGF, hypopharyngeal squamous cell carcinoma, tumorigenic phenotypes, AKT-mTOR-VEGF signaling, epithelial-mesenchymal transition

## Abstract

**Background:**

Hypopharyngeal squamous cell carcinoma (HSCC), an aggressive HNSCC subtype characterized by high metastatic potential and poor prognosis, frequently overexpresses hepatoma-derived growth factor (HDGF), a factor implicated in tumor progression. This study investigates the functional role of HDGF in HSCC and its regulatory mechanisms involving epithelial-mesenchymal transition (EMT) and the AKT/mTOR/VEGF signaling pathway.

**Methods:**

Bioinformatic analysis of TCGA data revealed elevated HDGF expression in HSCC tissues, significantly correlating with clinical stage. HDGF expression was depleted in the FaDu HSCC cell line using siRNA. Cell proliferation, migration, and invasion were assessed using CCK-8, wound healing, and Transwell assays, respectively. Western blotting evaluated changes in EMT markers (E-cadherin, N-cadherin, Snail, Slug) and key components of the AKT/mTOR/VEGF pathway (p-AKT, p-mTOR, VEGFA).

**Results:**

Bioinformatics analysis confirmed HDGF overexpression across HNSCC subtypes. In FaDu HSCC cells, siRNA-mediated HDGF knockdown significantly attenuated proliferation, migration, and invasion. Mechanistically, HDGF depletion reversed EMT progression, evidenced by E-cadherin upregulation and concurrent N-cadherin, Snail, and Slug downregulation. Western blotting demonstrated that HDGF knockdown suppressed AKT/mTOR signaling, as indicated by reduced p-AKT and p-mTOR levels, and decreased VEGFA expression.

**Conclusion:**

Our findings establish HDGF as a key promoter of HSCC progression through dual regulation of EMT and AKT/mTOR/VEGF pathways, suggesting its potential as a therapeutic target. These results provide mechanistic insights for developing HDGF-targeted strategies against this lethal malignancy, warranting further clinical exploration.

## Introduction

Head and neck squamous cell carcinoma (HNSCC) ranks as the sixth most common malignancy worldwide, encompassing cancers arising from the squamous epithelium of the oral cavity, oropharynx, larynx, and hypopharynx (1). Among these, hypopharyngeal squamous cell carcinoma (HSCC) is particularly aggressive and represents one of the most lethal HNSCC subtypes (2). Although surgery remains the primary treatment, outcomes are often poor and frequently result in functional impairment. Despite multimodal therapy combining surgery, chemotherapy, and radiotherapy, recurrence rates remain high, with up to 80% of patients developing cervical metastases after initial surgery and neck dissection. Emerging evidence suggests molecular targeted therapy as a promising approach for HSCC, highlighting the crucial need for identifying novel biomarkers.

Hepatoma-derived growth factor (HDGF), an acidic heparin-binding growth factor, has been implicated in the progression of diverse human malignancies, including hepatocellular carcinoma (3, 4), pancreatic cancer, esophageal cancer, gastric cancer (5) gastric cancer (6–8), colorectal cancer (9–11), and gastrointestinal stromal tumor (12). HDGF expression is significantly elevated in tumor tissues compared to adjacent non-tumorous tissues in cancers such as hepatocellular carcinoma and colorectal cancer. Furthermore, high HDGF expression correlates with poor prognosis in patients with hepatocellular carcinoma, pancreatic cancer, cholangiocarcinoma, gallbladder adenocarcinoma, and esophageal cancer (10). HDGF promotes proliferation, migration, and invasion in colorectal cancer, prostate cancer, and bladder cancer (13). However, the role of HDGF in HSCC progression remains unexplored.

This study aimed to investigate the functional role and underlying mechanisms of HDGF in HSCC progression. We performed HDGF knockdown in FaDu HSCC cells and examined its effects on cell proliferation, migration, and invasion *in vitro*. We further investigated alterations in the epithelial-mesenchymal transition (EMT) process and the AKT/mTOR/VEGF signaling pathway following HDGF depletion to elucidate its regulatory mechanisms in HSCC tumorigenic phenotypes.

## Materials and methods

### Bioinformatic analysis of HDGF in HSCC

HDGF mRNA expression in head and neck cancer was assessed using The Cancer Genome Atlas (TCGA) datasets. Differential HDGF expression between normal and HSCC tumor tissues was analyzed. Additionally, HDGF mRNA expression levels and their prognostic significance in HNSCC were explored using the Gene Expression Profiling Interactive Analysis (GEPIA) server.

### Cell culture​

The human FaDu HSCC cell line was obtained from the American Type Cell Collection (ATCC; Manassas, VA, USA). Cells were maintained in RPMI 1640 medium (Gibco, USA) supplemented with 10% fetal bovine serum (FBS; Gibco, USA), 100 U/ml penicillin (Sigma-Aldrich, St Louis, MO), and 100 μg/ml streptomycin (Sigma-Aldrich, St Louis, MO) at 37°C in a humidified atmosphere with 5% CO_2_.

### Lentiviral transduction​

Recombinant lentivirus carrying short hairpin RNA (shRNA) targeting HDGF and a control lentivirus were purchased from Bio-Link (Shanghai, China). Cellular transduction was performed according to the manufacturer’s protocol. Stable transductants were selected using puromycin (Solarbio, Beijing, China). The shRNA-HDGF target sequence was 5’-AACCGGCAGAAGGAGTACAAA-3’, while the scrambled control sequence was 5’-TTCTCCGAACGTGTCACGT-3’ (14).

### Quantitative real-time PCR

Total RNA was isolated using TRIzol reagent (Invitrogen, Carlsbad, CA, USA). Subsequently, 1 μg of RNA was reverse-transcribed into complementary DNA (cDNA) using the ReverTra Ace qPCR RT Kit (TOYOBO, Osaka, Japan). qRT-PCR was performed using SYBR Green Realtime PCR Master Mix (TOYOBO) on a QuantStudio 5 Real-Time PCR System (Thermo Fisher Scientific, Waltham, MA, USA). Primers were as follows: HDGF forward 5’-CTCTTCCCTTACGAGGAATCCA-3’, reverse 5’-CCTTGACAGTAGGGTTGTTCTC-3’; β-actin forward 5’-CATGTACGTTGCTATCCAGGC-3’, reverse 5’-CTCCTTAATGTCACGCACGAT-3’. HDGF mRNA expression levels were calculated using the 2-ΔΔCt method normalized to β-actin.

### CCK-8 cell proliferation assay​

Stably transduced FaDu cells were seeded into 96-well plates at 2000 cells/well. Cell viability was assessed after 1, 2, 3, or 4 days using the CCK-8 solution (Dojindo, Kumamoto, Japan). Absorbance was measured at 450 nm using a Microplate Reader (Bio-Rad Laboratories Inc, Hercules, CA, USA) after 1.5 hours of incubation. Experiments were performed in quintuplicate and repeated three times.

### Colony formation assay

Stably transduced FaDu cells were seeded in 6-well plates at 800 cells/well. After 14 days, colonies were fixed with 4% paraformaldehyde for 30 minutes and stained with 0.1% crystal violet for 15 minutes. Colonies containing >50 cells were counted. Experiments were performed in triplicate and repeated three times.

### Wound healing assay​

Cells were seeded in 6-well plates and cultured to 70%-80% confluency. A linear scratch was created using a 200 μl pipette tip. After washing with PBS to remove debris, wound closure was monitored at 0, 12, and 24 hours using an inverted microscope. The wound area was quantified using ImageJ software to calculate the migration ratio. Experiments were performed in triplicate.

### Transwell assay

Cell migration and invasion were assessed using Transwell chambers (8-μm pore size, Costar, New York, NY) (15). For migration, cells (3 × 10^4^/well) were seeded in the upper chamber in serum-free medium; the lower chamber contained medium with 20% FBS. For invasion, the upper chamber was pre-coated with Matrigel (1:8 dilution; BD Bioscience, San Jose, CA, USA). After 24 hours at 37°C with 5% CO2, migrated/invaded cells on the lower membrane surface were fixed, stained with crystal violet, and counted in five random high-power fields (×200). Experiments were performed in triplicate and repeated three times.

### Western blot assay

Total cellular protein was extracted using RIPA buffer (Beyotime, Shanghai, China) supplemented with 1% 100 mM PMSF (Solarbio, Beijing, China). Western blotting was performed as described previously (16). Primary antibodies used were: β-actin (1:1000, Abcam, Cambridge, MA, USA), HDGF (17), E-cadherin, N-cadherin, Snail, Slug (all 1:1000, CST, Boston, USA), VEGFA, p-AKT, p-mTOR (all 1:1000, Abcam). Membranes were incubated with HRP-conjugated secondary antibodies (1:1000, CST) and visualized using Western Blotting Luminol Reagent (Santa Cruz, CA, USA). Experiments were performed in triplicate.

### Statistical analysis

Data are presented as mean ± SD. Statistical analyses were performed using SPSS 22.0 (IBM, USA) and GraphPad Prism 9 (GraphPad Software, USA). Two-way ANOVA was used for CCK-8 assay data. Student’s t-test was used for qRT-PCR, colony formation, migration, and invasion assays. A p-value < 0.05 was considered statistically significant.

## Results

### HDGF is overexpressed in hypopharyngeal squamous cell carcinoma​

**Figure 1:**
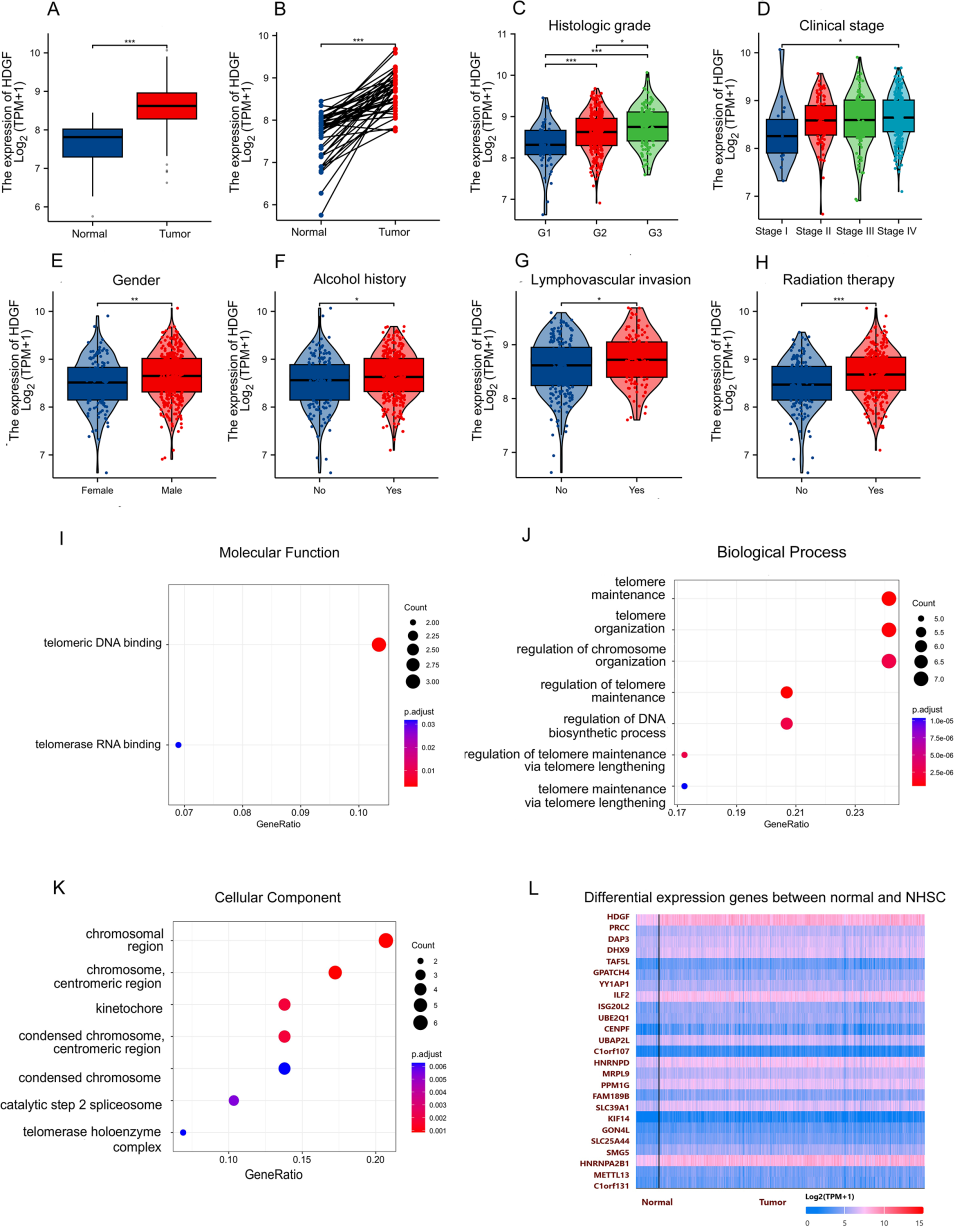
Expression characteristics and functional enrichment of HDGF in hypopharyngeal squamous cell carcinoma (HSCC). **(A)** HDGF expression was significantly higher in HSCC tissues than in normal tissues (*P* < 0.001). **(B)** Paired analysis confirmed HDGF upregulation in tumors compared with matched normal samples (*P* < 0.001). **(C)** HDGF expression increased with histologic grade and was highest in G3 tumors (*P* < 0.001). **(D)** HDGF levels elevated with clinical stage and were higher in stage IV than stage I (*P* < 0.05). **(E)** HDGF expression was higher in male patients. **(G)** HDGF levels were elevated in patients with lymphovascular invasion (*P* < 0.05). **(H)** Patients with a history of radiotherapy showed higher HDGF expression (*P* < 0.05). **(I)** GO Molecular Function analysis revealed enrichment in telomeric DNA binding and telomerase RNA binding. **(J)** GO Biological Process terms included telomere maintenance, telomere organization, and regulation of chromosomal organization. **(K)** GO Cellular Component terms included chromosomal region, centromeric region, kinetochore, condensed chromosome, and telomerase holoenzyme complex. **(L)** Heatmap of differentially expressed genes (DEGs) between normal and HSCC tissues. Data are presented as mean ± SD. *P < 0.05, **P < 0.01, ***P < 0.001, ****P < 0.0001.

Analysis of TCGA data revealed that HDGF mRNA expression was significantly higher in HSCC tumor tissues compared to normal tissues in both unpaired and paired samples (**Figures 1A, B**). HDGF expression increased with higher pathological grade (**Figure 1C**) and advanced clinical stage (**Figure 1D**). Database correlation analysis showed elevated HDGF expression in males and individuals with a history of alcohol abuse (**Figures 1E, F**). Furthermore, HDGF expression was increased in cases with lymphovascular invasion and following radiation therapy (**Figures 1G, H**). GO annotation analysis of the top 30 proteins most correlated with HDGF in HSCC implicated these proteins in telomeric DNA binding (molecular function), telomere maintenance (biological process), and chromosomal regions (cellular component) (**Figures 1I–K**) (**Supplementary table 1**). HDGF showed strong gene expression correlations in both normal and HSCC tissues (**Figure 1L**).

### HDGF knockdown suppresses FaDu cell proliferation and colony formation​

**Figure 2:**
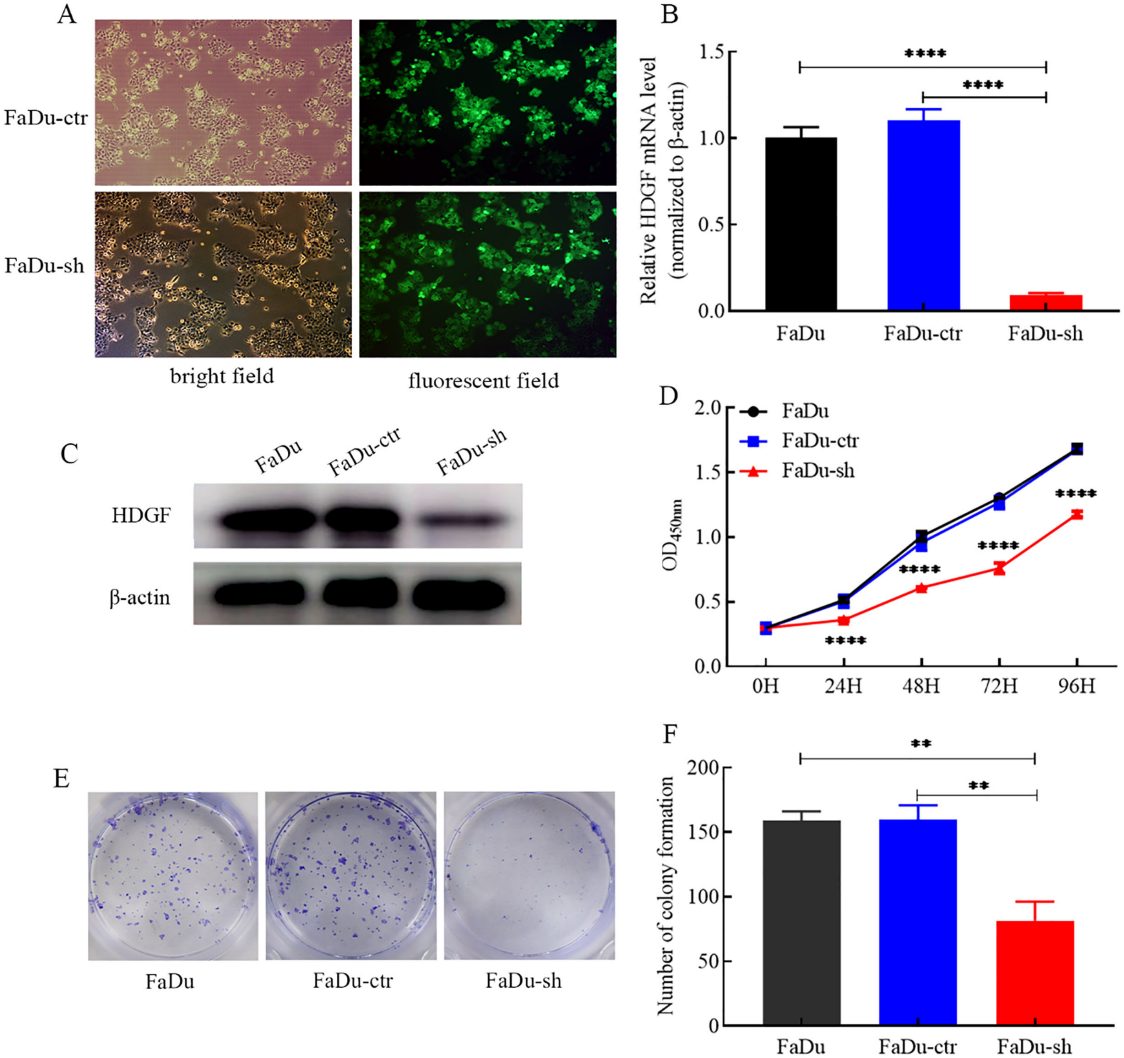
Effects of HDGF knockdown on proliferation and colony formation of FaDu cells. **(A)** Lentiviral transduction efficiency (>80% GFP-positive cells; magnification, ×200).**(B)** Relative HDGF mRNA expression in FaDu cells after transduction.**(C)** HDGF protein expression in FaDu cells after transduction. **(D)** CCK-8 assay showing reduced proliferation of FaDu cells after HDGF knockdown.**(E, F)** Colony formation assay demonstrating reduced colony formation capacity after HDGF knockdown. Data are mean ± SD; *P < 0.05, **P < 0.01, ***P < 0.001,****P < 0.0001.

HDGF knockdown in FaDu cells was achieved using lentiviral shRNA. Transduction efficiency exceeded 80%, confirmed by GFP expression (**Figure 2A**). Both HDGF mRNA (**Figure 2B**) and protein (**Figure 2C**) levels were significantly reduced. The CCK-8 assay demonstrated that HDGF knockdown markedly suppressed FaDu cell proliferation compared to control cells (**Figure 2D**). Similarly, the colony formation assay revealed a significant reduction in colony-forming capacity following HDGF knockdown (**Figures 2E, F**). These results indicate that HDGF critically regulates FaDu cell proliferation and clonogenicity.

### HDGF depletion inhibits FaDu cell migration and invasion​

**Figure 3:**
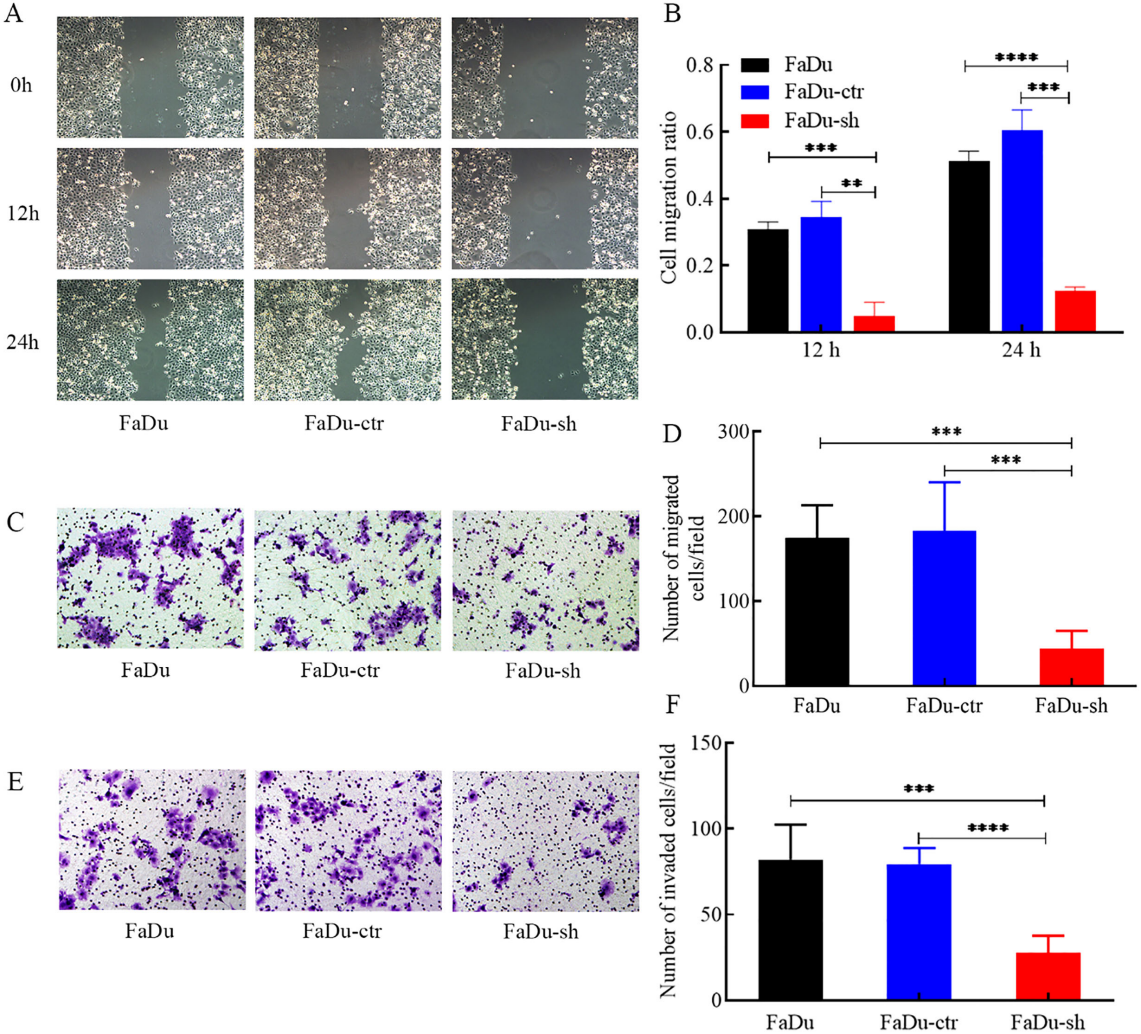
Effects of HDGF depletion on migration and invasion of FaDu cells. **(A, B)** Wound healing assay (representative images at x100 magnification). **(C, D)** Transwell migration assay (representative images at x100 magnification). **(E, F)** Transwell Matrigel invasion assay (representative images at x100 magnification). HDGF knockdown significantly reduced migration **(A-D)** and invasion **(E, F)** capacities. Data are mean ± SD; *P < 0.05, **P < 0.01, ***P < 0.001,****P < 0.0001.

Wound healing assays showed a significant reduction in the migration ratio of FaDu cells after HDGF knockdown (**Figures 3A, B**). Transwell migration assays confirmed a marked inhibition of migratory ability (**Figures 3C, D**). Furthermore, Transwell Matrigel invasion assays demonstrated that HDGF depletion significantly suppressed the invasive capacity of FaDu cells (**Figures 3E, F**). These findings highlight the essential role of HDGF in regulating FaDu cell migration and invasion.

### HDGF knockdown inhibits EMT in FaDu cells​

**Figure 4:**
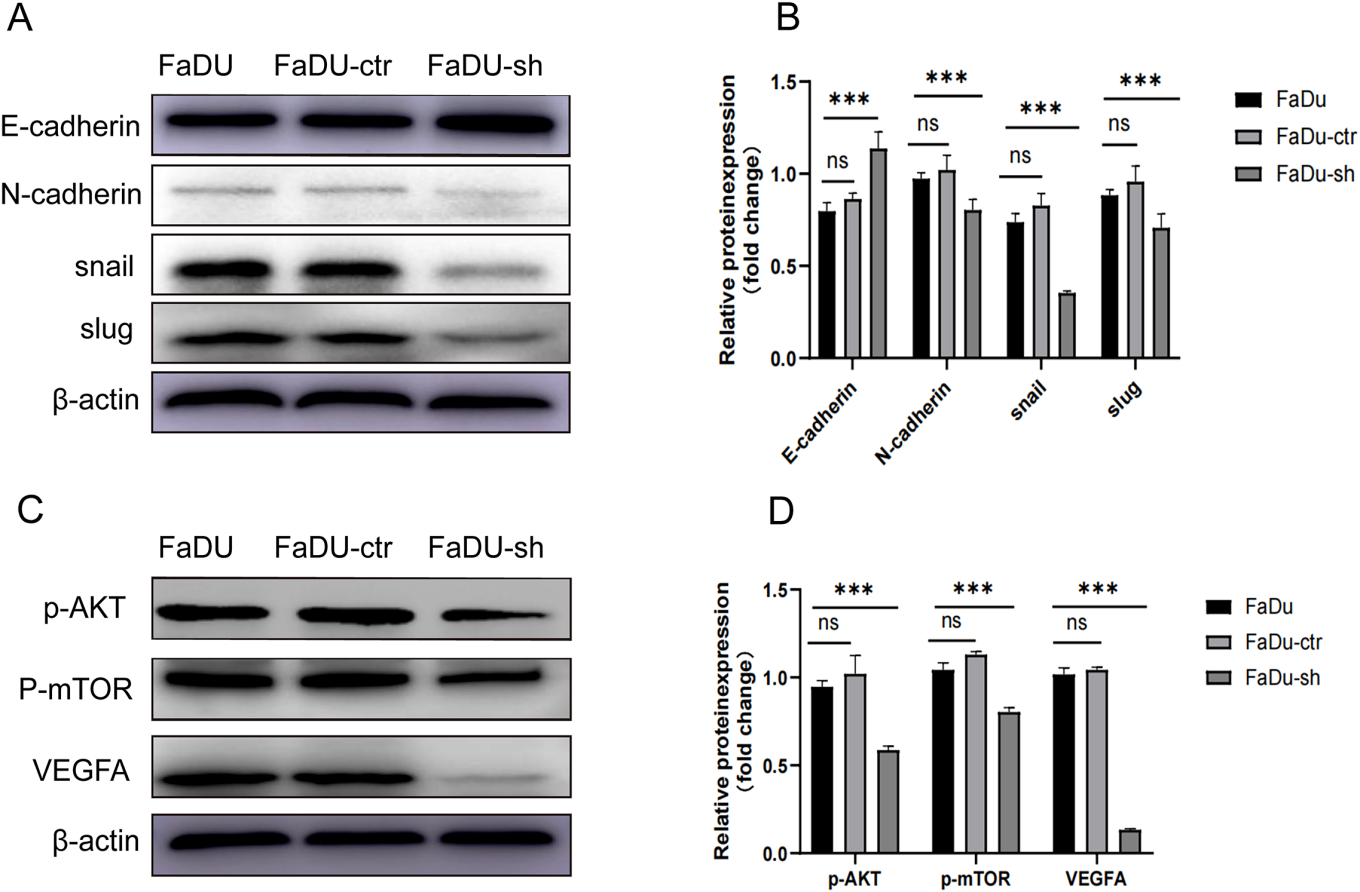
Effects of HDGF knockdown on EMT and AKT/mTOR/VEGF signaling in FaDu cells. **(A, B)** Western blot analysis of EMT-related proteins: N-cadherin, Snail, and Slug decreased; E-cadherin increased after HDGF knockdown. **(C, D)** Western blot analysis of AKT/mTOR/VEGF pathway components: p-AKT, p-mTOR, and VEGFA decreased after HDGF knockdown. Data are mean ± SD; *P < 0.05, **P < 0.01, ***P < 0.001,****P < 0.0001.

Western blot analysis revealed that HDGF knockdown in FaDu cells significantly decreased the expression of the mesenchymal marker N-cadherin and the EMT transcription factors Snail and Slug, while increasing the expression of the epithelial marker E-cadherin (**Figures 4A, B**). These changes indicate that HDGF depletion reverses EMT progression.

### HDGF knockdown suppresses the AKT/mTOR/VEGF pathway in FaDu cells​

Western blot analysis demonstrated that HDGF knockdown in FaDu cells significantly decreased the phosphorylation levels of AKT (p-AKT) and mTOR (p-mTOR), and suppressed VEGFA expression (**Figures 4C, D**). These findings suggest that HDGF promotes tumor progression by activating the AKT/mTOR/VEGF signaling pathway.

## Discussion

Hypopharyngeal squamous cell carcinoma (HSCC) is an aggressive HNSCC subtype characterized by high regional metastasis rates and poor prognosis. The molecular mechanisms driving its aggressive behavior remain incompletely understood, hindering effective treatment development. Hepatoma-derived growth factor (HDGF) has emerged as a key oncogenic factor in various cancers (12), promoting tumor growth, angiogenesis, and metastasis. However, its specific role in HSCC progression was previously undefined.

Our bioinformatic analysis of TCGA data confirmed HDGF overexpression in HSCC tissues compared to normal tissues, and revealed associations with higher grade, advanced stage, male gender, alcohol abuse, lymphovascular invasion, and post-radiation status (**Figure 1**). Functional studies employing siRNA-mediated HDGF knockdown in FaDu HSCC cells demonstrated its critical role in promoting malignant phenotypes. *In vitro* assays revealed that HDGF depletion significantly attenuated proliferation, migration, and invasion (**Figure 3**), aligning with reports linking HDGF to poor prognosis in head and neck carcinomas (18) and extending its mechanistic role specifically to HSCC.

Mechanistically, we provide evidence that HDGF depletion suppresses EMT progression, as evidenced by increased E-cadherin and decreased N-cadherin, Snail, and Slug expression (**Figure 4A**). This reversion from a mesenchymal to a more epithelial state is associated with reduced tumor aggressiveness (19). Furthermore, we identified that HDGF significantly impacts the AKT/mTOR/VEGF signaling pathway (**Figure 4B**). HDGF knockdown suppressed AKT and mTOR phosphorylation and downregulated VEGFA expression. This pathway is central to promoting cell survival, proliferation, metabolism (20), and angiogenesis, processes crucial for HSCC growth and metastasis. Our findings are consistent with HDGF’s role in activating growth and angiogenic pathways in other cancers (21).

This study has limitations. First, findings are primarily based on *in vitro* cell line models, which may not fully recapitulate the complex *in vivo* HSCC microenvironment. Second, while HDGF’s role in the AKT/mTOR/VEGF pathway was established, potential crosstalk with other signaling cascades remains unexplored. Future research should address these aspects for a more comprehensive understanding.

Notably, recent preclinical studies on anti-HDGF antibodies have provided robust support for our conclusion that “HDGF is an important therapeutic target.” Research has demonstrated that in non-small cell lung cancer (NSCLC) xenograft models, HDGF-specific monoclonal antibodies (such as HDGF-C1 and HDGF-H3) significantly inhibit tumor growth. It is particularly noteworthy that in EGFR-mutant NSCLC models, the combination of anti-HDGF antibodies and osimertinib not only achieved complete or near-complete tumor regression but also markedly prolonged progression-free survival (22, 23). The underlying mechanism may involve synergistic inhibition of the AKT/mTOR and MAPK pathways—which were also found to be activated in our study (24, 25). Furthermore, anti-HDGF antibodies exhibited significant efficacy in pancreatic cancer models, with no observable toxicity across all experimental models (25). These cross-cancer evidences robustly validate the therapeutic value of targeting HDGF, laying a solid foundation for developing mono- or combination therapies involving anti-HDGF antibodies for the treatment of HSCC.

This study has certain limitations. Firstly, the conclusions are primarily derived from *in vitro* cell models, which cannot fully recapitulate the complex tumor microenvironment *in vivo*. Therefore, validating the tumor-promoting role of HDGF using nude mouse xenograft models represents our primary follow-up objective. Secondly, although this study provides key evidence supporting HDGF’s regulation of the AKT/mTOR/VEGF pathway, more comprehensive mechanistic verification, such as pathway rescue experiments, remains to be conducted in the future. Furthermore, exploring the upstream regulators and broader downstream effector networks of HDGF will be essential for a comprehensive understanding of its oncogenic mechanisms.

In conclusion, our study establishes HDGF as a key promoter of HSCC progression by demonstrating its critical role in driving proliferation, migration, invasion, EMT, and activation of the AKT/mTOR/VEGF pathway. These findings strongly suggest HDGF as a promising therapeutic target for HSCC. Developing HDGF-specific inhibitors (e.g., small molecules or antibodies) or incorporating them into combination therapies holds potential for improving the prognosis of patients with this aggressive malignancy.

## Data Availability

The original contributions presented in the study are included in the article/**Supplementary Material**. Further inquiries can be directed to the corresponding author.
